# Host transcriptome-guided drug repurposing for COVID-19 treatment: a meta-analysis based approach

**DOI:** 10.7717/peerj.9357

**Published:** 2020-06-10

**Authors:** Tamizhini Loganathan, Srimathy Ramachandran, Prakash Shankaran, Devipriya Nagarajan, Suma Mohan S

**Affiliations:** School of Chemical & Biotechnology, SASTRA Deemed to be University, Thanjavur, India

**Keywords:** COVID-19, SARS-CoV-2, Drug repurposing, Host transcriptome

## Abstract

**Background:**

Coronavirus disease 2019 (COVID-19) caused by severe acute respiratory syndrome coronavirus 2 (SARS-CoV-2) has been declared a pandemic by the World Health Organization, and the identification of effective therapeutic strategy is a need of the hour to combat SARS-CoV-2 infection. In this scenario, the drug repurposing approach is widely used for the rapid identification of potential drugs against SARS-CoV-2, considering viral and host factors.

**Methods:**

We adopted a host transcriptome-based drug repurposing strategy utilizing the publicly available high throughput gene expression data on SARS-CoV-2 and other respiratory infection viruses. Based on the consistency in expression status of host factors in different cell types and previous evidence reported in the literature, pro-viral factors of SARS-CoV-2 identified and subject to drug repurposing analysis based on DrugBank and Connectivity Map (CMap) using the web tool, CLUE.

**Results:**

The upregulated pro-viral factors such as *TYMP*, *PTGS2*, *C1S*, *CFB*, *IFI44*, *XAF1*, *CXCL2*, and *CXCL3* were identified in early infection models of SARS-CoV-2. By further analysis of the drug-perturbed expression profiles in the connectivity map, 27 drugs that can reverse the expression of pro-viral factors were identified, and importantly, twelve of them reported to have anti-viral activity. The direct inhibition of the *PTGS2* gene product can be considered as another therapeutic strategy for SARS-CoV-2 infection and could suggest six approved PTGS2 inhibitor drugs for the treatment of COVID-19. The computational study could propose candidate repurposable drugs against COVID-19, and further experimental studies are required for validation.

## Introduction

COVID-19 is a pulmonary syndrome caused by a novel strain of coronavirus, and according to the World Health Organization report as on 13th April 2020, 1,773,084 people have been infected with about 111,650 deaths globally (Coronavirus Disease, 2019). The primary mode of SARS CoV-2 transmission is through respiratory droplets generated during coughing and sneezing by infected patients (Huang et al., 2020). Symptoms include dry cough, fatigue myalgia, fever, and dyspnea; however, the disease progresses to severe illness and leads to death in 6% of confirmed cases due to massive alveolar damage and progressive respiratory failure (Rothe et al., 2020). Coronaviruses possess the largest genomes (26.4–31.7 kb) of all RNA viruses encoding four main structural proteins contain spike (S), membrane (M), envelope (E), and nucleocapsid (N) proteins. After the virus enters the host cell, the genome is transcribed and then translated (Cascella et al., 2020). Invading respiratory viruses would either suppress or evade the innate immune and adaptive responses on the host’s side and increase virulence, which leads to disease outcome.

With its limited genome size, the virus extensively utilizes the host factors for their replication via inducing alterations in the host gene expression resulting in modulated immune response (De Wilde et al., 2018). Therefore, the transcriptome analysis of host cell upon virus infection is useful for identifying host immune response dynamics and also significant host factors that would facilitate virus infection. To develop effective therapeutic strategies, it is necessary to understand the expression of host factors upon SARS-CoV-2 infection. According to the current treatment scenario, drug repurposing from FDA approved drugs would be an effective alternative method that would improve the host factor against SARS-CoV-2 virus infection. The transcriptome guided drug repurposing approaches utilize the drug perturbed expression profiles to identify potential drug candidates, which show anti-correlation with the disease signature (Arakelyan et al., 2019). In the present study, initially, we attempted to conduct the transcriptome analyses by utilizing the publicly available host transcriptome profiles against SARS-CoV-2 and also other respiratory virus infections that could provide information on altered host factors upon infection. Next, based on the identified SARS-CoV-2 induced pro-viral host factors, drug repurposing analyses were performed to identify the possible drugs for the treatment of the pandemic infection caused by COVID-19, which would help to decrease the mortality of COVID-19 patients globally.

## Materials and Methods

### Data retrieval and sample selection

**Figure 1:**
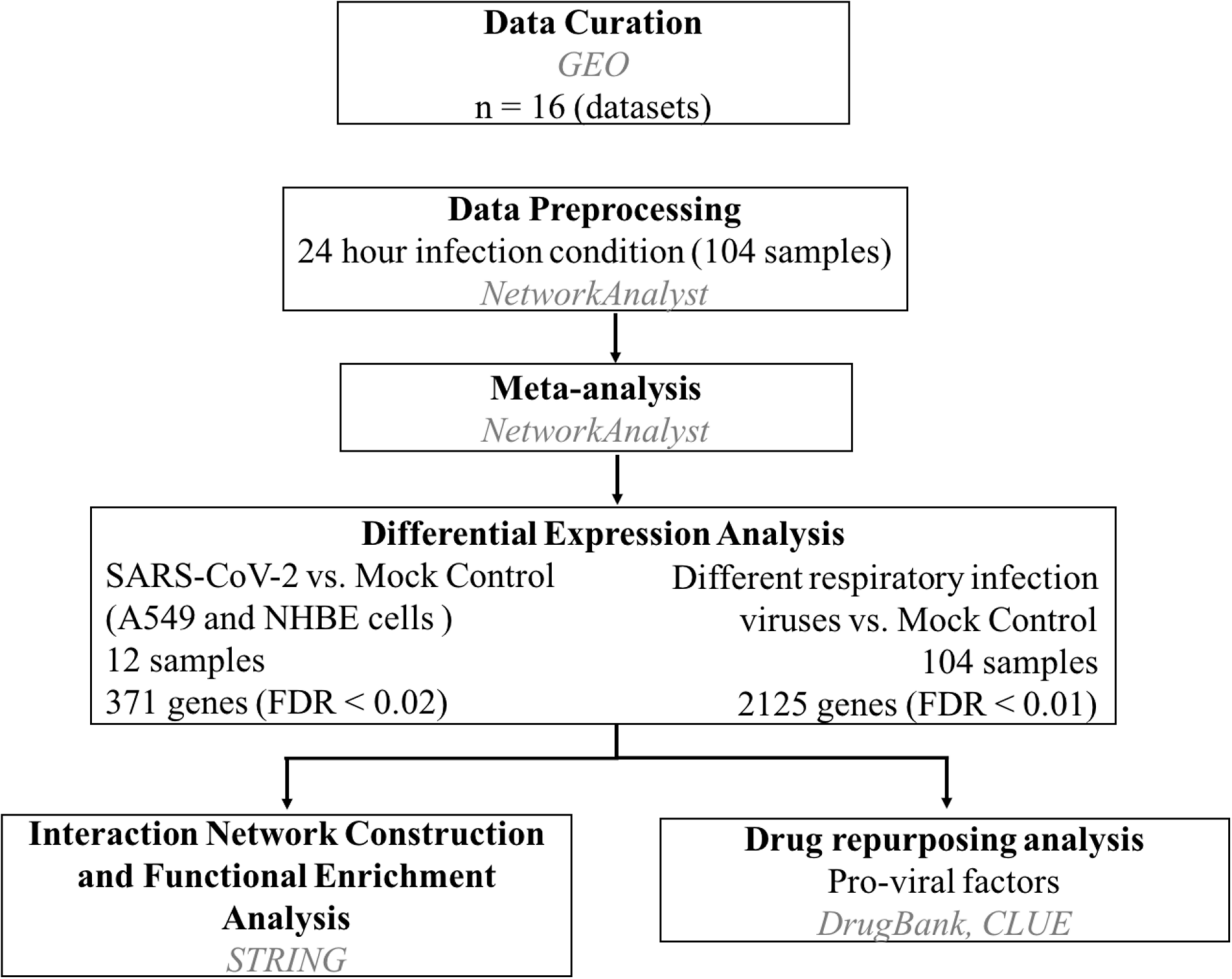
Work flow to identify host pro-viral factors and drug repurposing.

Gene expression profile datasets based on expression profiling by high-throughput sequencing and microarray in response to different coronaviruses such as Middle East respiratory syndrome coronavirus (MERS-CoV), SARS-CoV-1 and SARS-CoV-2 infection in the human host were retrieved from Gene Expression Omnibus (GEO) database (http://www.ncbi.nlm.nih.gov/geo/) and OmicsDI (https://www.omicsdi.org/). The datasets with coronavirus infected group and mock control group were included in the study. Sixteen datasets were obtained, and three of them were based on expression profiling by high-throughput sequencing (RNA-Seq), and the remaining thirteen were from microarray experiments. The cell types of the identified datasets are human airway epithelium cells (HAE), Calu-3 lung adenocarcinoma, primary fibroblasts, primary human microvascular endothelial cells, primary human dendritic cells. The only available transcriptome profile dataset on SARS-CoV-2 (as of 9th April 2020) (GSE147507) was based on the expression profiling in 24-h infected cells (Blanco-Melo et al., 2020). So we have selected only samples from 24-h infected conditions and the corresponding mock controls for the meta-analysis. Based on the above criteria, thirteen datasets (GSE147507, GSE17400, GSE45042, GSE47962, GSE33267, GSE37827, GSE122876, GSE100496, GSE86528, GSE100509, GSE79172, GSE81909, GSE48142) were selected for the meta-analysis. A total of 104 samples from the thirteen datasets selected for the meta-analysis (Table S1). Among the 104 samples, 52 samples belong to respiratory virus-infected condition, and 52 of them were mock control samples. The overall workflow implemented in the study is reported in Fig. 1.

### Data processing and statistical meta-analysis

The preprocessing and statistical meta-analysis of the selected 104 samples were performed using the web-based tool NetworkAnalyst (Zhou et al., 2015). The identifiers of transcripts from high throughput sequencing and different microarray platforms were converted to Entrez gene IDs. The preprocessed microarray and RNA-Seq datasets were normalized using the log2 transformation. All the datasets passed the integrity check and subjected to study-specific batch effect adjustment using the ComBat methods in the Network Analyst tool. The Density plot and PCA-3D plot before and after the batch-effect adjustment indicate the proper incorporation of the datasets (Fig. S1). A total of 9,836 features from 104 samples consists of different respiratory virus infection models with two experimental conditions, 24-h infected condition and mock control were subjected to analysis. The meta-analysis was performed based on combining effect sizes method using the Fixed Effect Model with the significance level 0.01 and the differentially expressed genes in 24 h infection conditions of different respiratory viruses vs. mock control were obtained.

The RNA-Seq dataset with the GEO ID GSE147507 was used to identify the genes altered in SARS-CoV-2 infected conditions. The dataset consists of host transcriptome profiling based on two cell lines, A549 and NHBE. The 24 h SARS-CoV-2 infected and mock control samples from the two cell lines were subjected to separate differential expression analysis using the Gene expression table option of Network Analyst. Each dataset consists of 6 samples corresponding to 24 h SARS-CoV-2 and mock-control. The gene expression table uploaded with the options, organism-human, data type-bulk RNA-seq and the gene level summarization-mean. There are 16,375 and 15,770 features selected for A549 and NHBE cells, respectively. The unannotated genes were filtered and log2-counts per million normalization applied. After normalization, the graphical outputs, box plots, PCA, and density plots were verified to check the quality of the data. After that, differential expression analysis in case vs. mock condition performed using the method, DESeq2 and the differentially expressed genes were obtained. The significant thresholds, adjusted *P*-value 0.02 and log2 fold change 0.3 were applied to identify the significantly differentially expressed genes. Among the significant genes, the genes showing consistent tendency in NHBE and A549 cells (31 genes) were selected for further analysis. Among the 31 genes, the pro and antiviral factors were identified based on the literature survey. The heatmap2 tool in Galaxy was used to generate the heat map of the differentially expressed genes.

### Protein interaction analysis and gene set enrichment analysis

Protein level interaction analysis was performed using the STRING Program (Szklarczyk et al., 2019). The selected differentially expressed genes were submitted to a multi-gene entry option in STRING to obtain the protein level interaction network. Cluster option was used to identify the cluster of interactions in the network and the obtained interaction details were used to construct a protein–protein interaction network using Cytoscape (Shannon et al., 2003). The Pathway and Gene Ontology enrichment details were obtained from STRING based on the FDR cut off <0.01.

### Drug repurposing analysis

The DrugBank Version 5.1.4 (http://www.drugbank.ca/) was used to obtain the drug-target link between existing drug molecules and the DEGs. The differentially expressed genes showing consistent tendency were subjected to target search in DrugBank. Only approved or experimental drug groups were included in the study.

Drug repurposing analysis was performed using the Connectivity Map using the web application CLUE (https://clue.io) (Subramanian et al., 2017). The query option in the tools was selected to find the perturbagens that give rise to opposing expression signature of the pro-viral factors identified in the study. The query requires a minimum of 10 genes, so 8 pro-viral factors (*TYMP*, *PTGS2*, *C1S*, *CFB*, *IFI44*, *XAF1*, *CXCL2* and *CXCL3*) showing consistent up-regulation in NHBE and A549 cells, and two pro-viral factors (*NFKB1* and *TLR2*) upregulated in NHBE cells were subjected to analysis. The up-regulated pro-viral factors were compared to each signature in the CMap reference (Gene expression (L1000) Touchstone) database. The heatmap of the connectivity score (*tau*) of perturbagens (2837 small-molecule compounds) was obtained. The top-scored compounds with CMap *tau* score <−99 with the highest anti-correlation with the upregulated ten pro-viral genes were identified.

## Results

### Identification of differentially expressed host genes with COVID-19 infection

**Figure 2:**
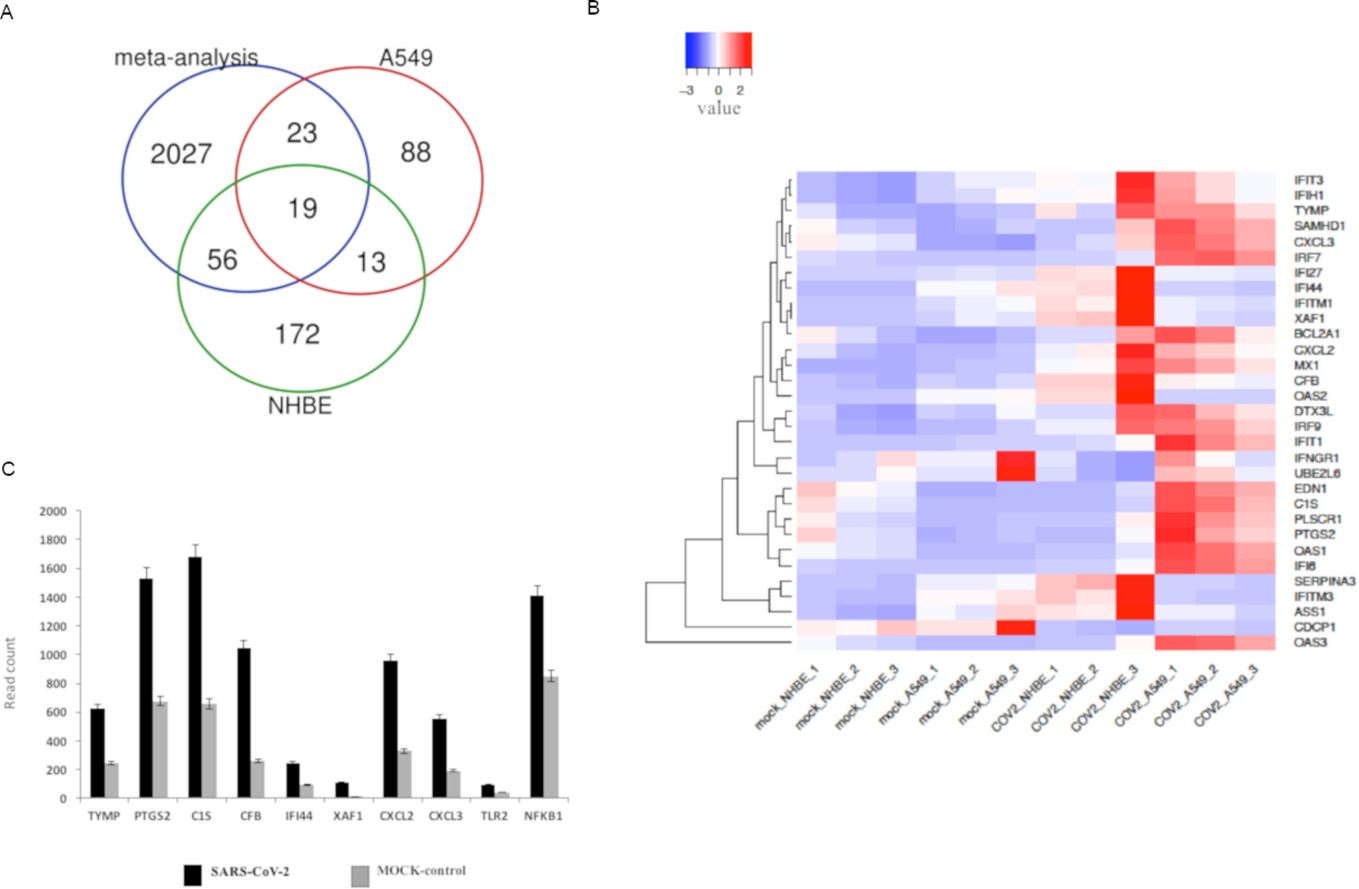
Significant host factors. (A) Venn diagram showing overlap in the differentially expressed genes in meta-analysis of different respiratory infection viruses and SARS-CoV-2 infection in NHBE and A549 cells. (B) Heat map of the 31 differentially expressed genes in SARS-CoV-2 vs. Mock control infection in A549 and NHBE cells. (C) Average expression levels of the 10 pro-viral factors in SARS-CoV-2 infected and mock control conditions from GEO dataset GSE147507.

In this study, the host factors in response to SARS-CoV-2 and other coronavirus infections were analyzed using a computational approach. A meta-analysis strategy utilized to identify differentially expressed genes common in the human host infection mediated by different respiratory infection viruses. The 16 datasets from gene expression profiling studies based on high-throughput sequencing and microarray experiments were obtained from GEO (Table S1). The publicly available host gene expression profiles of respiratory infection viruses till 9th April 2020 was used for the analysis. The dataset on SARS-CoV-2 (GEO ID: GSE147507) consists of 24-h infected and the mock-control samples of primary human lung epithelium (NHBE) and transformed lung alveolar(A549) cells (Blanco-Melo et al., 2020). A total of 104 samples (52 respiratory virus-infected and 52 mock control samples) selected for the meta-analysis considering only 24 h infected samples from different datasets consisting of respiratory virus-infected human host models of SARS-CoV-2, SARS-CoV, MERS-CoV, and Respiratory syncytial virus (RSV). The differential expression analysis of various respiratory infection viruses vs. mock-control by meta-analysis reported 2,125 genes based on the FDR < 0.01 (Table S2). Next, the differentially expressed genes, specifically in SARS-CoV-2 infected conditions, were identified in A549 and NHBE cells. The Table S3 reports 143 and 260 differentially expressed genes in A549 and NHBE cells, respectively, based on the adjusted *P* value cutoff < 0.02. Together in NHBE and A549 cells, a total of 371 unique genes were reported as differentially expressed (Table S3). The Venn diagram reports the overlap of the common genes in a meta-analysis of different respiratory virus infection vs. mock and SARS-CoV-2 vs. mock conditions in 24 h infection models (Fig. 2A). Only 19 differentially expressed genes were found to be common in the meta-analysis of different respiratory infection viruses and SARS-CoV-2 specific analysis in different cell lines, which indicate SARS-CoV-2 specific gene signatures in a 24 h host infection models. The Venn diagram reports 32 genes common between A549 and NHBE cells (highlighted in Table S3). Among that, 31 genes noticed to be upregulated in both NHBE and A549 cells. The remaining one gene, KRT4 found to be down-regulated in A549 and upregulated in NHBE cells. The 31 genes showing consistent upregulation tendency in SARS-CoV-2 infected NHBE, and A549 cells were selected for further analysis (Table 1). Among that, 19 genes overlapping with the meta-analysis data (Fig. 2A) observed to have the same upregulation tendency and are included in Table 1. Therefore, the 19 genes are common in 24-h infection models of the different respiratory infection viruses, and that includes *IFI6, IFIT1, MX1, IRF9, IRF7, OAS1, IFIH1, IFI27, PLSCR1, OAS2, IFITM1, CXCL2, IFI44, PTGS2, BCL2A1, CXCL3, XAF1*, and *EDN1*. The heat map of the thirty-one significant genes was reported in Fig. 2B and has shown the same tendency (Table 1) in the original analysis reported by Blanco-Melo et al. (2020), though the statistical parameters found to vary.

**Table 1:** Significant host genes in SARS-CoV-2 infection. List of 31 differentially expressed genes in SARS-CoV-2 infection showing consistent expression pattern in A549 and NHBE cell types. The pro-viral factors are highlighted by cell shading.

Sl. No.	Symbol	Gene name	A549		NHBE		Meta-analysis	
			log2FC	Adj. *P*	log2FC	Adj. *P* Val.	Combined effect size	*P*-val
1	IFI6	interferon alpha inducible protein 6	3.4726	2.20E−285	0.4910	9.25E−03	0.8919	2.64E−03
2	IFIT1	interferon induced protein with tetratricopeptide repeats 1	3.0171	1.33E−164	0.5381	1.24E−02	1.9102	2.66E−07
3	MX1	MX dynamin like GTPase 1	3.1067	1.05E−158	1.7708	5.10E−34	1.3564	5.01E−05
4	IRF9	interferon regulatory factor 9	1.8037	1.65E−83	1.1033	5.56E−26	1.8136	1.80E−07
5	IRF7	interferon regulatory factor 7	2.1793	1.35E−79	0.7363	1.76E−04	1.0570	1.15E−03
6	OAS1	2′-5′-oligoadenylate synthetase 1	1.3695	1.18E−65	1.2423	1.99E−17	0.8506	6.66E–03
7	OAS3	2′-5′-oligoadenylate synthetase 3	1.2382	1.85E−55	1.1097	1.93E−20	–	–
8	DTX3L	deltex E3 ubiquitin ligase 3L	1.1582	3.05E−34	0.3844	2.84E−03	–	–
9	IFIT3	interferon induced protein with tetratricopeptide repeats 3	1.3826	9.21E−32	0.5429	3.26E−03	–	–
10	IFIH1	interferon induced with helicase C domain 1	1.2169	7.46E−23	0.5538	2.43E−04	1.2175	3.67E−04
11	IFI27	interferon alpha inducible protein 27	1.2853	2.45E−22	1.1013	9.88E−11	1.5037	2.34E−07
12	PLSCR1	phospholipid scramblase 1	0.9179	1.85E−22	0.7025	2.22E−07	0.8592	8.45E−03
13	SAMHD1	SAM and HD domain containing deoxynucleoside triphosphate triphosphohydrolase 1	0.8025	1.39E−14	0.5916	9.18E−04	–	–
14	CFB	complement factor B	0.8597	6.60E−14	1.5625	2.80E−41	–	–
15	C1S	complement C1s	0.6360	5.81E−13	0.5241	1.70E−02	–	–
16	OAS2	2′-5′-oligoadenylate synthetase 2	0.7404	5.53E−09	1.0724	1.40E−19	0.7975	6.80E−03
17	IFITM1	interferon induced transmembrane protein 1	0.6736	8.72E−08	1.0275	8.18E−10	0.8890	6.18E−03
18	CXCL2	C-X-C motif chemokine ligand 2	0.5591	4.12E−07	1.1055	1.85E−16	4.1305	0.00E+00
19	IFI44	interferon induced protein 44	0.5678	4.09E−06	0.7175	3.88E−07	1.2257	1.02E−04
20	TYMP	thymidine phosphorylase	0.5890	5.64E−06	0.7536	5.59E−06	–	–
21	PTGS2	prostaglandin-endoperoxide synthase 2	0.5111	5.26E−05	0.4438	9.36E−03	1.7201	1.46E−08
22	IFITM3	interferon induced transmembrane protein 3	0.5781	1.32E−04	0.6595	3.63E−07	–	–
23	BCL2A1	BCL2 related protein A1	0.5339	1.44E−03	1.1167	1.07E−10	0.9892	3.70E−04
24	CXCL3	C-X-C motif chemokine ligand 3	0.4599	1.44E−03	1.2931	4.08E−15	2.3068	1.71E−10
25	SERPINA3	serpin family A member 3	0.4451	4.84E−03	1.1934	1.13E−20	–	–
26	XAF1	XIAP associated factor 1	0.3157	5.75E−03	1.2454	1.83E−13	1.2515	7.89E−05
27	ASS1	argininosuccinate synthase 1	0.3673	8.11E−03	0.4619	4.14E−05	–	–
28	EDN1	endothelin 1	0.3148	8.45E−03	0.8201	3.97E−08	1.0609	1.32E−03
29	UBE2L6	ubiquitin conjugating enzyme E2 L6	0.63038	3.76E−09	0.35748	0.019644	–	–
30	IFNGR1	interferon gamma receptor 1	0.38229	0.015405	0.36279	0.017155	–	–
31	CDCP1	CUB domain containing protein 1	0.33301	0.012564	0.34665	0.0012701	–	–

The genes upregulated in SARS-CoV-2 mainly included interferon-inducible genes. The genes *MX1* (MX dynamin-like GTPase 1), *IRF9* (interferon regulatory factor 9), *OAS1*, *OAS3* (2′–5′-oligoadenylate synthetase) and *IFI27* (interferon-alpha inducible protein 27) which showed consistently more than two-fold upregulation in SARS-CoV-2 infected A549 and NHBE cells (Table 1). Among the 31 significantly upregulated genes, eight genes were found to be pro-viral host factors required for efficient viral replication, which included *TYMP*, *PTGS2*, *C1S*, *CFB*, *IFI44*, *XAF1*, *CXCL2* and *CXCL3* and were further considered for drug repurposing analysis. The average expression level of the reported pro-viral factors in SARS-CoV-2 infected and mock-control conditions are shown in Fig. 2C.

### Functional annotation and pathway enrichment of significant host genes in SARS-CoV-2 infection

**Figure 3:**
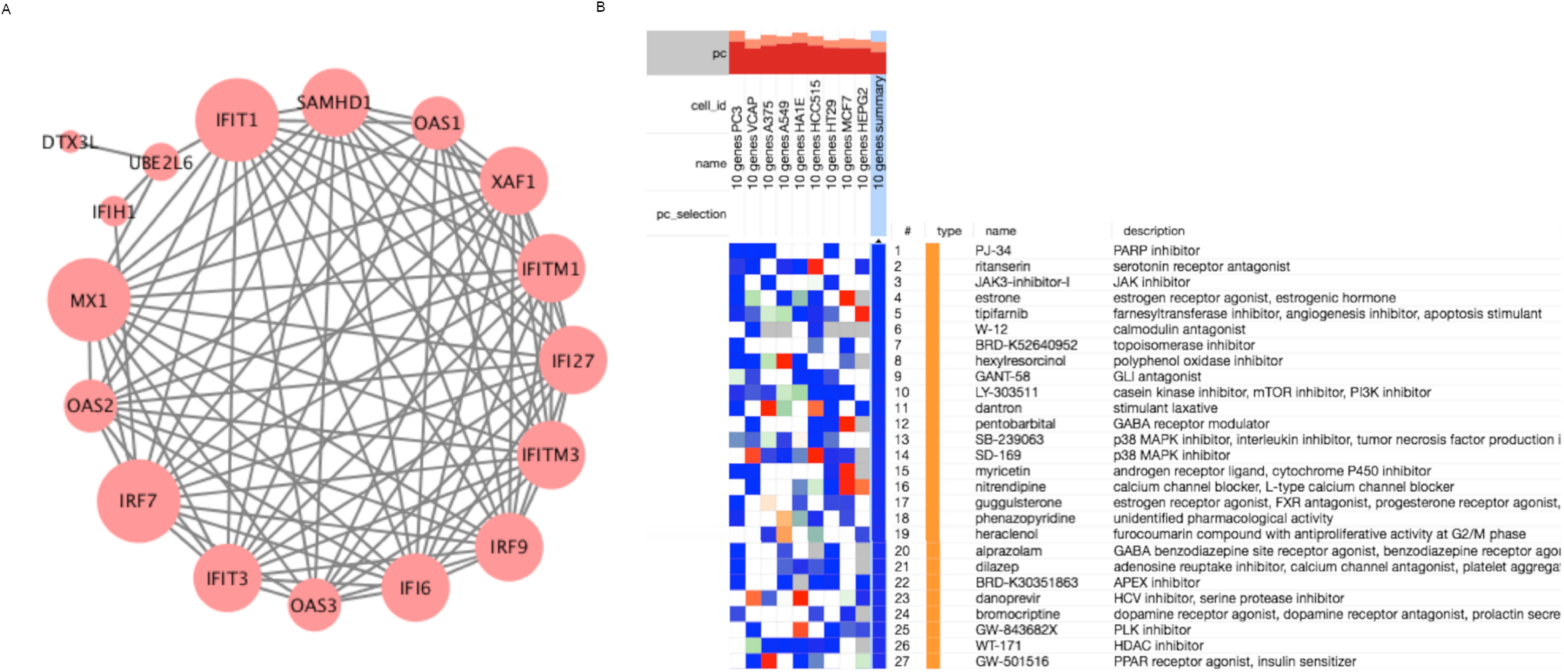
Protein level interaction cluster and CMap analysis details for the host factors. (A) Protein interaction cluster formed by the upregulated genes of SARS-CoV-2 infection. (B) Heat map of the top selected compounds from CMap based analysis using CLUE.

The selected 31 genes reported in Table 1 were subjected to protein level interaction and functional enrichment analysis using the web tool, STRING. The protein–protein interaction network was constructed mainly considering interactions with high confidence score >0.9. A significant network with 94 edges was obtained with a PPI enrichment *P*-value: < 1.0E−16. The fourteen nodes of the network formed a cluster with a dense overlap of 88 edges, and those nodes included, *IFI6, IFI27, IFIT1, IFIT3, IFITM1, IFITM3, IRF7, IRF9, IFIH1, SAMHD1, OAS1, OAS2, OAS3 MX1* and *XAF1* and were reported in Fig. 3A. The node size in the network indicates the connectivity degree, and the gene *IRF7* was found to have the highest degree of 14. The cluster forming genes are part of the Interferon alpha/beta signaling, and importantly, the pathway enrichment analysis based on the REACTOME pathway reports this signaling as the top enriched pathway with a significant FDR of 2.32E−24 and enriched with 14 genes (Table 2). The other significant pathways are cytokine signaling in the immune system enriched with 16 genes, the immune System pathway with 20 genes and the Antiviral mechanism by IFN-stimulated gens with six enriched genes. The top enriched pathways and biological processes were reported in Table 2. The other relevant pathways from KEGG and REACTOME enriched by the set of genes were reported in Table S4.

**Table 2:** Functional annotation of host genes. Top enriched pathways and biological processes of the selected 31 genes in SARS-CoV-2 infection.

#Term ID	Term description	Observed gene count	FDR	Proteins
Reactome pathway				
HSA-909733	Interferon alpha/beta signaling	14	2.32E−24	IFI27, IFI6, IFIT1, IFIT3, IFITM1, IFITM3, IRF7, IRF9, MX1, OAS1, OAS2, OAS3, SAMHD1, XAF1
HSA-913531	Interferon Signaling	16	1.28E−22	IFI27, IFI6, IFIT1, IFIT3, IFITM1, IFITM3, IFNGR1, IRF7, IRF9, MX1, OAS1, OAS2, OAS3, SAMHD1, UBE2L6, XAF1
HSA-1280215	Cytokine Signaling in Immune system	18	1.34E−17	CXCL2, IFI27, IFI6, IFIT1, IFIT3, IFITM1, IFITM3, IFNGR1, IRF7, IRF9, MX1, OAS1, OAS2, OAS3, PTGS2, SAMHD1, UBE2L6, XAF1
HSA-168256	Immune System	22	1.39E−14	C1S, CFB, CXCL2, DTX3L, IFI27, IFI6, IFIH1, IFIT1, IFIT3, IFITM1, IFITM3, IFNGR1, IRF7, IRF9, MX1, OAS1, OAS2, OAS3, PTGS2, SAMHD1, UBE2L6, XAF1
HSA-1169410	Antiviral mechanism by IFN-stimulated genes	6	5.59E−08	IFIT1, MX1, OAS1, OAS2, OAS3, UBE2L6
Biological process				
GO:0006952	Defense response	26	1.41E−23	ASS1, C1S, CFB, CXCL2, CXCL3, DTX3L, EDN1, GIG25, IFI27, IFI6, IFIH1, IFIT1, IFIT3, IFITM1, IFITM3, IFNGR1, IRF7, IRF9, MX1, OAS1, OAS2, OAS3, PLSCR1, PTGS2, SAMHD1, XAF1
GO:0060337	Type I interferon signaling pathway	14	1.56E−23	IFI27, IFI6, IFIT1, IFIT3, IFITM1, IFITM3, IRF7, IRF9, MX1, OAS1, OAS2, OAS3, SAMHD1, XAF1
GO:0045087	Innate immune response	21	1.56E−23	ASS1, C1S, CFB, DTX3L, EDN1, IFI27, IFI6, IFIH1, IFIT1, IFIT3, IFITM1, IFITM3, IFNGR1, IRF7, IRF9, MX1, OAS1, OAS2, OAS3, SAMHD1, XAF1
GO:0009615	Response to virus	16	1.51E−19	DTX3L, IFI44, IFIH1, IFIT1, IFIT3, IFITM1, IFITM3, IFNGR1, IRF7, IRF9, MX1, OAS1, OAS2, OAS3, PLSCR1, SAMHD1
GO:0051707	Response to other organism	21	1.51E-19	ASS1, CXCL2, CXCL3, DTX3L, EDN1, IFI44, IFIH1, IFIT1, IFIT3, IFITM1, IFITM3, IFNGR1, IRF7, IRF9, MX1, OAS1, OAS2, OAS3, PLSCR1, PTGS2, SAMHD1

The Gene Ontology (GO) enrichment analysis of the 31 genes was reported in Table S4. The top five biological processes include defense response, type I interferon signaling pathway, innate immune response, response to the virus, and response to other organisms. The enriched molecular functions are 2′–5′-oligoadenylate synthetase activity and double-stranded RNA binding (Table S4).

### Drug repurposing against SARS-CoV-2

To find potential drug molecules for repurposing against SARS-CoV-2 infection, we have done the DrugBank search with the 31 upregulated genes. Drug groups with approved or experimental status were considered for the analysis, and the identified drug molecules for the target genes were tabulated in Table 3. Approved or experimental drugs were identified as modulators of the protein product of three genes, *CFB*, *ASS1, IFNGR1* and *PTGS2*. The pharmacological action unknown for drugs targeting CFB and ASS1 and a protein binder reported for IFNGR1. Importantly, PTGS2 is a pro-viral factor, and 76 approved drug molecules identified as inhibitor/antagonist of PTGS2 protein (Table 3).

**Table 3:** Repurposable drugs from DrugBank. List of drugs targeting pro-viral factors from DrugBank.

Protein	DrugBank ID	Name	Drug group	Pharmacological action	Actions	tau_score	Therapy area
CFB	DB02459	4-guanidinobenzoic acid	experimental	unknown		–	not available
	DB04491	Diisopropylphosphono Group	experimental	unknown		–	not available
PTGS2	DB00159	Icosapent	approved, nutraceutical	Yes	Inhibitor	16.07	Treating for depression
	DB00480	Lenalidomide	approved	unknown	negative modulator	−75.35	maintenance therapy (anti-inflammatory drug)
	DB00482	Celecoxib	approved, investigational	Yes	Inhibitor	−78.25	anti-inflammatory drug
	DB00605	Sulindac	approved, investigational	Yes	Inhibitor	69.63	anti-inflammatory agent
	DB00712	Flurbiprofen	approved, investigational	Yes	Inhibitor	10.62	Nonsteroidal anti-inflammatory drugs
	DB00784	Mefenamic acid	approved	Yes	Inhibitor	86.16	Nonsteroidal anti-inflammatory drugs
	DB00812	Phenylbutazone	approved, vet_approved	Yes	Inhibitor	−59.87	nonsteroidal anti-inflammatory drug
	DB00991	Oxaprozin	approved	Yes	Inhibitor	−25.36	nonsteroidal anti-inflammatory drug
	DB00244	Mesalazine	approved	Yes	Inhibitor	27.96	aminosalicylate and anti-inflammatory.
	DB00316	Acetaminophen	approved	Yes	Inhibitor	–	opioid pain medication
	DB00328	Indomethacin	approved, investigational	Yes	Inhibitor	–	nonsteroidal anti-inflammatory drug
	DB00461	Nabumetone	approved	Yes	Inhibitor	76.11	nonsteroidal anti-inflammatory drug
	DB00465	Ketorolac	approved	Yes	Inhibitor	39.76	nonsteroidal anti-inflammatory drug
	DB00469	Tenoxicam	approved	Yes	Inhibitor	−78.71	antiinflammatory agent with analgesic and antipyretic properties
	DB00500	Tolmetin	approved	Yes	Inhibitor	94.88	nonsteroidal anti-inflammatory drug
	DB00573	Fenoprofen	approved	Yes	Inhibitor	–	antiinflammatory and antipyretic properties
	DB00586	Diclofenac	approved, vet_approved	Yes	Inhibitor	−44.57	nonsteroidal anti-inflammatory drug
	DB00749	Etodolac	approved, investigational, vet_approved	Yes	inhibitor	49.63	anti-inflammatory agent with analgesic and antipyretic properties
	DB00788	Naproxen	approved, vet_approved	Yes	inhibitor	−72.61	nonsteroidal anti-inflammatory drug
	DB00814	Meloxicam	approved, vet_approved	Yes	inhibitor	16.48	nonsteroidal anti-inflammatory drug
	DB00861	Diflunisal	approved, investigational	Yes	inhibitor	−15.47	anti-inflammatory and antipyretic properties
	DB00939	Meclofenamic acid	approved, vet_approved	Yes	inhibitor	−85.24	nonsteroidal agent
	DB00945	Acetylsalicylic acid	approved, vet_approved	Yes	inhibitor	–	Cardiovascular
	DB00963	Bromfenac	approved	Yes	inhibitor	66.6	nonsteroidal anti-inflammatory drug (NSAID)
	DB01009	Ketoprofen	approved, vet_approved	Yes	inhibitor	−45.36	nonsteroidal anti-inflammatory agent (NSAIA)
	DB01014	Balsalazide	approved, investigational	Yes	inhibitor	−63.18	anti-inflammatory drug
	DB01050	Ibuprofen	approved	Yes	inhibitor	96.49	non-steroidal anti-inflammatory drug (NSAID)
	DB01283	Lumiracoxib	approved, investigational	Yes	inhibitor	–	nonsteroidal anti-inflammatory drug.
	DB01399	Salsalate	approved	Yes	inhibitor	–	nonsteroidal anti-inflammatory drug.
	DB00936	Salicylic acid	approved, investigational, vet_approved	Yes	inhibitor	–	non-steroidal anti-inflammatory drug (NSAID)
	DB01041	Thalidomide	approved, investigational, withdrawn	unknown	antagonist	23.43	Immunomodulatory drug
	DB01404	Ginseng	approved, investigational, nutraceutical	unknown	inhibitor	–	not available
	DB01628	Etoricoxib	approved, investigational	Yes	inhibitor	–	non-steroidal anti-inflammatory drugs
	DB01600	Tiaprofenic acid	approved	Yes	inhibitor	33.41	non-steroidal anti-inflammatory drug
	DB02266	Flufenamic acid	approved	unknown		−62.8	not available
	DB00554	Piroxicam	approved, investigational	Yes	inhibitor	−71.6	nonsteroidal anti-inflammatory drugs (NSAIDs)
	DB00250	Dapsone	approved, investigational	unknown	substrate	−61.99	anti-inflammatory immunosuppressive properties as well as antibacterial and antibiotic properties
	DB06725	Lornoxicam	approved, investigational	Yes	inhibitor	–	non-steroidal anti-inflammatory drug (NSAID)
	DB06802	Nepafenac	approved, investigational	unknown	inhibitor	–	non-steroidal anti-inflammatory drug (NSAID)
	DB00795	Sulfasalazine	approved	Yes	inhibitor	−93.8	anti-inflammatory drug
	DB01435	Antipyrine	approved, investigational	unknown	inhibitor	–	analgesic and antipyretic
	DB01419	Antrafenine	approved	unknown	inhibitor	–	anti-inflammatory effect
	DB01401	Choline magnesium trisalicylate	approved	unknown	inhibitor	–	non-steroidal anti-inflammatory drug (NSAID)
	DB00233	Aminosalicylic acid	approved	unknown	inhibitor	–	anti-mycobacterial agent
	DB00963	Bromfenac	approved	unknown	inhibitor	66.6	non-steroidal anti-inflammatory drug (NSAID)
	DB00887	Bumetanide	approved	unknown	inducer	61.14	Cardiovascular
	DB06774	Capsaicin	approved	unknown	inhibitor	39.94	anti-fungal agent
	DB00856	Chlorphenesin	approved, experimental	unknown	inhibitor	–	antifungal and some antibacterial properties
	DB00720	Clodronic acid	approved, investigational, vet_approved	unknown	inhibitor	–	antihypercalcemic agent
	DB00035	Desmopressin	approved	unknown	inducer	–	analgesic effect
	DB01395	Drospirenone	approved	unknown	inhibitorinducer	–	antiandrogen effects
	DB00515	Cisplatin	approved	unknown	inhibitor	–	chemotherapy medication
	DB00884	Risedronic acid	approved, investigational	unknown	inducer	–	
	DB00360	Sapropterin	approved, investigational	unknown	inducer	–	Cardiovascular
	DB00041	Aldesleukin	approved	unknown	inducer	–	
	DB00620	Triamcinolone	approved, vet_approved	unknown	inhibitor	87.19	generic medication.
	DB08819	Tafluprost	approved	unknown	inducer	–	
	DB08910	Pomalidomide	approved	Yes	inhibitor	–	
	DB00773	Etoposide	approved	unknown	substrate	98.52	
	DB08439	Parecoxib	approved	Yes	inhibitor	20.25	
	DB09213	Dexibuprofen	approved, investigational	Yes	inhibitor	–	
	DB11133	Omega-3 fatty acids	approved, nutraceutical	unknown	substrate	–	
	DB06736	Aceclofenac	approved, investigational	Yes	inhibitor	–	
	DB13783	Acemetacin	approved, investigational	Yes	antagonist	–	
	DB09212	Loxoprofen	approved	Yes	antagonist	−80.04	non-steroidal anti-inflammatory drug
	DB09216	Tolfenamic acid	approved, investigational	Yes	antagonist	–	non-steroidal anti-inflammatory drug
	DB09214	Dexketoprofen	approved, investigational	unknown	antagonist	99.54	non-steroidal anti-inflammatory drug
	DB09214	Dexketoprofen	approved, investigational	unknown	inhibitor	99.54	non-steroidal anti-inflammatory drug
	DB11079	Trolamine salicylate	approved	Yes	inhibitor	–	
	DB11071	Phenyl salicylate	approved	unknown	antagonist	–	
	DB11327	Dipyrithione	approved	unknown		–	
	DB13961	Fish oil	approved, nutraceutical	Yes	inhibitor	–	
	DB11323	Glycol salicylate	approved	Yes	antagonist	–	
	DB11201	Menthyl salicylate	approved	Yes	antagonist	–	
	DB00210	Adapalene	approved	Yes	inhibitor	–	
	DB09061	Cannabidiol	approved, investigational	unknown	inhibitor	–	
ASS1	DB00128	Aspartic acid	approved, nutraceutical	unknown		–	
	DB00155	Citrulline	approved, investigational, nutraceutical	unknown		–	
IFNGR1	DB00033	Interferon gamma-1b	approved, investigational	yes	binder	–	Chronic granulomatous disease and Osteopetrosis

**Figure 4:**
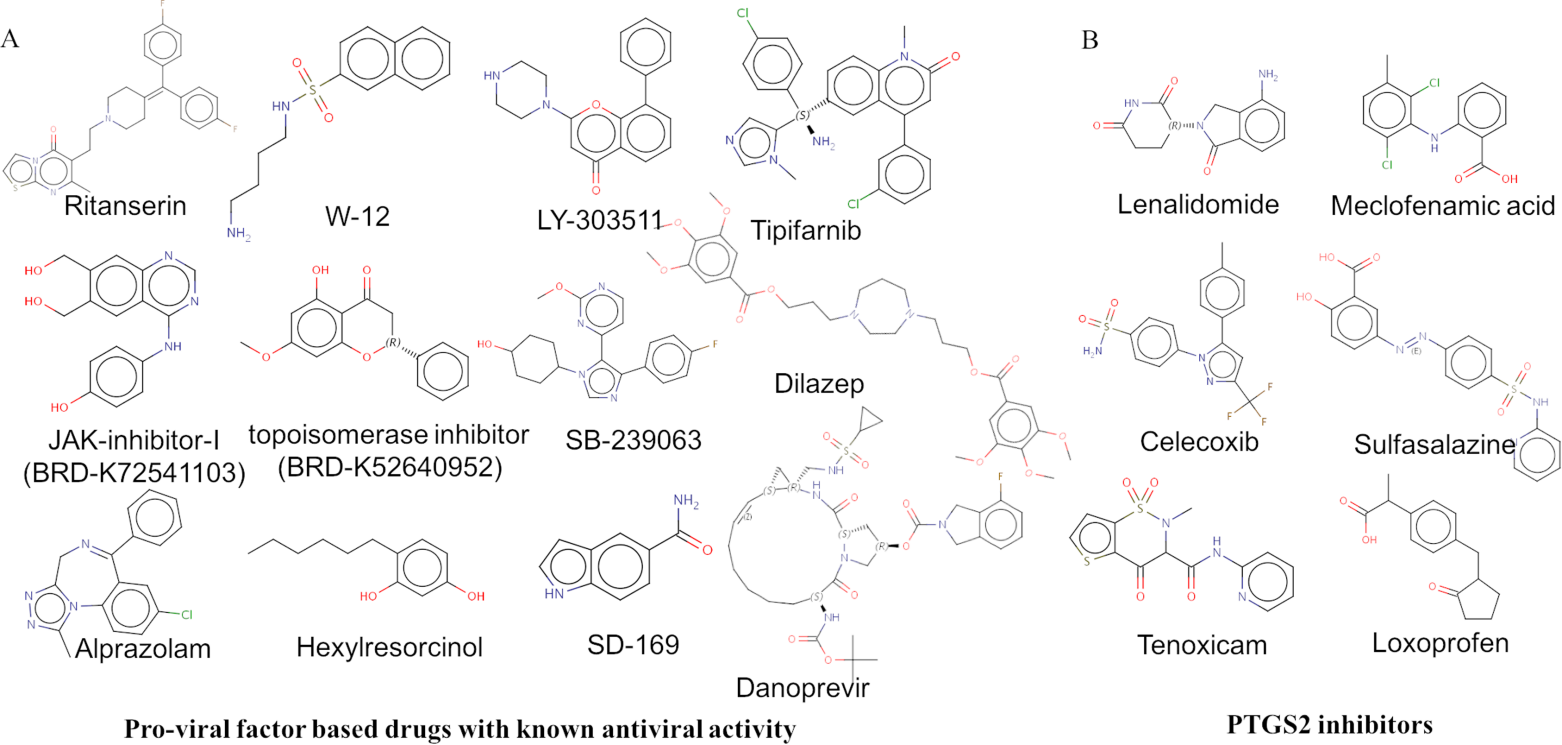
Repurposable drugs from the study. (A) Suggested drug candidates for COVID-19 treatment by drug repurposing approach based on CMap. (B) PTGS2 inhibitors.

Next, we have utilized a gene expression signature-based drug repurposing strategy based on CMap (Connectivity Map) using the web tool, CLUE. A gene set can be queried to drug perturbation signatures to identify and rank drugs according to the similarity in gene expression. A positive score indicates the similarity and negative score indicate the reverse effect of the drug signatures with the queried genes, and its magnitude contributes to the magnitude of similarity. Among the 28 significantly upregulated genes, eight genes were found to be pro-viral factors that are important for the viral infection and need to be targeted for the therapy point of view. Along with the eight genes, *NFKB1* and *TLR2* also found to be pro-viral and upregulated in SARS-CoV-2 infected NHBE cells. Therefore, the ten upregulated genes, *TYMP, PTGS2, C1S, CFB, IFI44, XAF1, CXCL2, CXCL3, NFKB1* and *TLR2* were subjected to connectivity map-based gene expression signature search to find therapeutic compounds. We could identify compounds with significantly correlated and anti-correlated signatures with that of the ten pro-viral host factors considered for the analysis. The top-scored compounds with CMap connectivity score (tau score) <−99 with the highest anti-correlation with the upregulated ten pro-viral genes are reported in Table 4. There were 27 compounds with significant anti-correlation, and the heat map obtained from CLUE analysis shown in Fig. 3B (Table 4). The approved drugs among those include estrone (estrogen receptor agonist), hexylresorcinol (polyphenol oxidase inhibitor), pentobarbital (GABA receptor modulator), nitrendipine (calcium channel blocker), phenazopyridine (targeting SCN1A), heraclenol (Cobamamide), alprazolam (benzodiazepine receptor agonist), bromocriptine (dopamine receptor agonist) and WT-171 (Vorinostat) (HDAC inhibitor). Twelve of the 27 top drugs found to have reported anti-viral activity against different viruses which includes, ritanserin, JAK3-inhibitor-I (BRD-K72541103), tipifarnib, W-12, Topoisomerase inhibitor (BRD-K52640952), hexylresorcinol, LY-303511, SB-239063, SD-169, alprazolam, dilazep, danoprevir and are highlighted in Table 4 and the structure of the drug molecules are shown in Fig. 4A. Next, we analyzed the potential impact of the reported 27 drugs on the natural defense against infection. For that, we have checked the correlation between drug-perturbed expression profile with the 23 anti-viral proteins reported from the study using the connectivity map. Among the 27 drugs obtained based on pro-viral factors, seven drugs, tipifarnib, dilazep, GW-843682X, estrone, myricetin, guggulsterone, bromocriptine reported having mean connectivity score <−90 and can down-regulate the anti-viral proteins. Therefore, the above mentioned seven drugs can impact the host natural defense against viral infection and therefore, caution needs to be taken.

**Table 4:** Repurposable drugs based on Connectivity Map. List of repurposable drugs based on connectivity map analysis using CLUE. Drugs with known anti-viral activity is highlighted in bold characters.

Sl. No.	Id	Name*	Description	Target	tau score	Drug bank id	Therapy area	Drug group	Anti-viral activity (PUBMED id)
1	BRD-K11853856	PJ-34	PARP inhibitor	EEF2, PARP1, PARP15, PARP3	−99.93		anticancer drugs		
2	BRD-K40887525	**Ritanserin**	serotonin receptor antagonist	HTR2A, HTR2C, HTR2B, ADRA1A, ADRA1B, ADRA1D, HTR1A, HTR1B, HTR1D, HTR5A, HTR6, HTR7	−99.89	DB12693	clinical trials for schizophrenia and migraine.	Investigational	19703243
3	BRD-K72541103	**JAK3-inhibitor-I**	JAK inhibitor	JAK3	−99.89				22359619
4	BRD-A37959677	Estrone	estrogen receptor agonist, estrogenic hormone	ESR1, ESR2	−99.86	DB00655	menopausal hormone therapy	Approved	
5	BRD-K63195589	**Tipifarnib**	farnesyltransferase inhibitor, angiogenesis inhibitor, apoptosis stimulant	FNTA, FNTB	−99.86	DB04960	cancer	Investigational	28677645
6	BRD-K78084463	**W-12**	calmodulin antagonist		−99.82		Cardiac dysrhythmias	unknown	27940224
7	BRD-K52640952	**BRD-K52640952**	topoisomerase inhibitor	TOP1	−99.72				1707642
8	BRD-K99946902	**hexylresorcinol**	polyphenol oxidase inhibitor	TYR	−99.61	DB11254	antiseptic, anthelmintic, and local anesthetic properties	Approved	26408353
9	BRD-K64451768	GANT-58	GLI antagonist	DHH, GLI1, IHH	−99.61				
10	BRD-K22385716	**LY-303511**	casein kinase inhibitor, mTOR inhibitor, PI3K inhibitor	CSNK2A1, CSNK2A2, CSNK2B, MTOR	−99.54				29369973, 30405592, 26388843
11	BRD-K10065684	Dantron	stimulant laxative		−99.51	DB04816	Drugs for Constipation	Approved, Investigational, Withdrawn	
12	BRD-A44448661	Pentobarbital	GABA receptor modulator	GABRA1, CHRNA4, CHRNA7, GABRA2, GABRA3, GABRA4, GABRA5, GABRA6, GABRB1, GABRB2, GABRB3, GABRD, GABRE, GABRG1, GABRG2, GABRG3, GABRP, GABRQ, GRIA2, GRIK2, GRIN1, GRIN2A, GRIN2B, GRIN2C, GRIN2D, GRIN3A, GRIN3B	−99.44	DB00312 (APRD01174)	anti-anxiety agent	Approved, Investigational, Vet approved	
13	BRD-K98143437	**SB-239063**	p38 MAPK inhibitor, interleukin inhibitor, tumor necrosis factor production inhibitor	MAPK11, MAPK14, PTGS2, TNF	−99.44				11046054
14	BRD-K91904471	**SD-169**	p38 MAPK inhibitor	MAPK14	−99.37				11046054
15	BRD-K43149758	Myricetin	androgen receptor ligand, cytochrome P450 inhibitor	PIK3CG, AR, CYP3A4	−99.37	DB02375 (EXPT02265)	antioxidant properties	Experimental	
16	BRD-A02006392	Nitrendipine	calcium channel blocker, L-type calcium channel blocker	CACNA1C, CACNA1D, CACNA2D1, CACNA1H, CACNA1S, CACNA2D2, CACNB2, CACNG1, KCNN4	−99.35	DB01054 (APRD00421)	antihypertensive agent	Approved, Investigational	
17	BRD-A59808129	Guggulsterone	estrogen receptor agonist, FXR antagonist, progesterone receptor agonist, cholesterol inhibitor, IKK inhibitor, PXR agonist	NR1H4, PGR, AR, ESR1, IKBKB, NR1I2, NR3C1, NR3C2	−99.33		endocrinology	pre-clinical	
18	BRD-K76304753	phenazopyridine	unidentified pharmacological activity	SCN1A	−99.22	DB01438	analgesic effect	Approved	
19	BRD-A77050075	heraclenol (Cobamamide)	furocoumarin compound with antiproliferative activity at G2/M phase		−99.22	DB11191	Neurological	Approved	347911148
20	BRD-K32398298	**Alprazolam**	GABA benzodiazepine site receptor agonist, benzodiazepine receptor agonist	GABRA1, GABRA2, GABRA3, GABRA5, CYP3A5, GABRA4, GABRA6, GABRB1, GABRB2, GABRB3, GABRD, GABRE, GABRG1, GABRG2, GABRG3, GABRP, GABRQ	−99.22	DB00404 (APRD00280, DB05925, DB05690)		Approved, Illicit, Investigational	8255908
21	BRD-K48722258	**Dilazep**	adenosine reuptake inhibitor, calcium channel antagonist, platelet aggregation inhibitor	SLC29A1, CACNA1C	−99.19	DB13715	cardiovascular agents	Experimental	25091947
22	BRD-K30351863	BRD-K30351863	APEX inhibitor	APEX1	−99.19				
23	BRD-K17823458	**Danoprevir**	HCV inhibitor, serine protease inhibitor		−99.15	DB11779 (DB06038)	protease inhibitor	Investigational	24295986
24	BRD-A60274948	Bromocriptine	dopamine receptor agonist, dopamine receptor antagonist, prolactin secretion inhibitor	DRD2, ADRA2A, ADRA2B, ADRA2C, DRD1, DRD3, DRD4, DRD5, HTR1A, HTR1B, HTR1D, HTR2A, HTR2B, HTR2C, ADRA1A, ADRA1B, ADRA1D, HTR6, HTR7, PRL	−99.08	DB01200 (APRD00622)	dopaminergic activity	Approved, Investigational	
25	BRD-K90382497	GW-843682X	PLK inhibitor	PLK1, PLK3	−99.05				
26	BRD-K74761218	WT-171 (Vorinostat)	HDAC inhibitor	HDAC6	−99.01	DB02546 (EXPT02902)	anti-coagulant activity	Approved, Investigational	5311
27	BRD-K14880289	GW-501516 (Cardarine)	PPAR receptor agonist, insulin sensitizer	PPARD, INS, PPARA	−99.01	DB05416 (DB13015)	cardiovascular	Investigational	9803963

We also checked the connectivity score of the PTGS2 inhibitors obtained from DrugBank (Table 3). Among the 76 drugs targeting PTGS2, six of them noticed to have a high negative tau score (tau score <−75), which indicates the possibility of reducing the expression of pro-viral factors (Table 3).Those drugs include lenalidomide (anti-inflammatory drug), celecoxib (anti-inflammatory drug), tenoxicam (anti-inflammatory agent with analgesic and antipyretic properties), meclofenamic acid (anti-inflammatory and antipyretic properties), Sulfasalazine (anti-inflammatory drug), loxoprofen(non-steroidal anti-inflammatory drug) (Fig. 4B). The anti-inflammatory drugs which target PTGS2, mefenamic acid, tolmetin, ibuprofen and dexketoprofen, the generic medication drug triamcinolone(generic medication) and the anti-cancer drug etoposide, found to report high positive tau score which indicates a high correlation with the expression level of pro-viral host factors noticed in the SARS-CoV-2 infection and the possibility of promoting the viral infection by the medication. Based on the current analysis, the potential PTGS2 inhibitors considered for drug repurposing against SARS-CoV-2 are reported in Fig. 4B.

## Discussion

A meta-analysis on publicly available gene expression profiles was adopted to identify respiratory virus infection mediated host response. We could identify common host genes in 24-h infection models of different respiratory infection viruses and SARS-CoV-2. A set of differentially expressed 31 genes showing consistent expression pattern in SARS-CoV-2 infected conditions in NHBE and A549 cells were identified. Apart from mouse epithelial cell lines, NHBE, and A549 are the commonly used human lung epithelial-derived cell lines to study the SARS coronaviruses (both classical and CoV-2) infection. Though A549 cells are susceptible to SARS-CoV infection, owing to the low expression level of the ACE2 receptor, this cell line is not highly permissive for SARS-CoV-2 and the infection rate is low (Harcourt et al., 2020). But, A549 cells supplemented with a vector expressing ACE2 enabled SARS-CoV-2 to replicate even in low infection conditions (Blanco-Melo et al., 2020). Based on the updates in the GEO dataset (GSE147507), the upregulated host factors common in both A549 cells expressing exogenous ACE2 receptor and Calu-3 cell lines in addition to NHBE cells are analyzed and there are nine common genes noticed, which includes *CXCL3, ASS1, UBE2L6, IRF7, C1S, SERPINA3, IRF9, IFI6 and CFB*. The present study is based on a limited number of datasets on SARS-CoV-2 and possible to have more candidate genes with more samples with increased sequencing depth. However, the study tried to pick the robust set of genes with consistent expression patterns in two different cell types. The pathway enrichment analysis highlights the Interferon alpha/beta signaling pathway, cytokine signaling, and immune system as the enriched pathways and defense and immune response associated process as the enriched biological processes, which is consistent with the host response observed in various virus-infected conditions.

During the initial stage of virus infection, the innate immune system will be stimulated to establish the first line of defense. Interferons (IFN) are a multigene family of inducible cytokines plays a critical role in initiating host antiviral responses (Boasso, 2013) and are commonly grouped as type I which includes IFN-α (leukocyte), IFN-β (fibroblast), and IFN-ω while type II IFN is also known as immune IFN (IFN-γ) (Schlaepfer et al., 2019). In most mild cases, type I IFN is highly effective at inhibiting viral replication during early short periods of viremia. However, in severe forms of viral replication, the ability of type I IFN to inhibit viral replication is overwhelmed (Long et al., 2009). Studies showed that in BALB/c mice infected with severe acute respiratory syndrome (SARS), there was a delay in Type I INF response leading to enhanced viral replication resulting in elevated lung cytokine/chemokine levels, vascular leakage, and impaired virus-specific T cell responses (Channappanavar et al., 2016).

IFN induces immune responses by activating the JAK/STAT pathway, which in turn forms a complex with interferon regulatory factor (IRF) and migrates to the nucleus in order to stimulate the expressions of over 300 IFN-stimulated genes (ISGs) that is necessary to inhibit viral replication (Teijaro, 2016). Among all ISGs, our analysis showed that IFN-α-inducible protein 6 (*IFI6*) and IFN-α-inducible protein 27 were elevated. In response to viral infection, among the IRF family members, particularly IRF-1, IRF-3, and IRF-7 are necessary for the production of type I IFN and also IFN-inducible genes (Bego, Mercier & Cohen, 2012). In our analyses, we also observed that there was an increase in IRF7 (log2FC=2.17) and IRF9 (log2FC = 1.80) during SARS-CoV-2 infection and also in other respiratory virus infection. Altogether, an increase in the level of IFN, IRFs, and ISGs plays a central role in antiviral responses, which might be during an early stage of infection, and we suggest that it could be used as a diagnostic marker upon virus infection like SARS-CoV-2. Moreover, in another study, it has been shown that the multiplication of SARS-CoV in cell culture can be strongly inhibited by pretreatment with interferon-beta (Spiegel et al., 2004). So, we could suggest that treatment with Interferonα/β mimics immediately after infection might stimulate the JAK/STAT pathway, which in turn stimulates ISGs and ultimately enhance antiviral effects.

Interferon-inducible transmembrane proteins (IFITMs) are identified as a key ISGs induced by interferon and interfere with virus entry. IFITMs (IFITM1/2/3/7) are induced by IFN and are necessary for innate immunity (Chen et al., 2019). IFITM2 and IFITM3 might reduce the infectivity of viruses by regulating virus-endosome fusion rates and accelerating the trafficking of virus-endosome to lysosomes (Spence et al., 2019). Similar to IFITM, 2′–5′-oligoadenylate synthetases (OAS1, OAS2, OAS3) are induced by type I IFN interferons and in the presence of viral dsRNA OAS catalyze oligomerisation of ATP to form 2′–5′-linked adenosine oligomers (2-5A), which would involve in the activation of RNase L degradative pathway that would cleave the viral RNA and ultimately control viral infection (Kristiansen et al., 2010; Leisching et al., 2019). In our analyses, we observed that there was up-regulation in IFITM (1 and 3) and OAS (1,2,3) in virus-infected cells, which could be due to the early stage of infection.

Interferon-induced protein with tetratricopeptide repeats (IFITs) is strongly induced by IFN-α/β are strong inducers, whereas IFN-γ acts as a weak inducer (Fensterl & Sen, 2011). IFIT3 is necessary for maintaining cell survival by decreasing the rate of apoptotic cell death. The knockdown of IFIT3 decreased the production of antiviral cytokine, IFN-β (Hsu et al., 2013). Our observation showed that there was an increased expression of IFIT 1 and 3 in virus-infected cells. Additionally, there was a significant increase in the anti-apoptotic protein, Bcl-2 related protein A, which might involve in protecting cell death against viral infection.

Interferon-induced with helicase C domain 1 *(IFIH1*) gene belonging to helicase family and encodes a cytoplasmic receptor critical for viral RNA sensing. During viral entry, IFIH interacts with viral RNA and leads to polymerization of IFIH1 molecules into a filament, which assembly assemble further to initiate signaling cascade to induce type 1 IFN production and leads to activation of antiviral genes (Asgari et al., 2017). The lSAM and HD domain-containing deoxynucleoside triphosphate triphosphohydrolase1 (SAMHD1) protein binds to viral RNA and exhibits exonuclease activity on single-stranded nucleic acids. Studies showed that the degradation of SAMHD1 might enhance the replication of herpes viruses in co-infected patients (Kim et al., 2013). MX dynamin-like GTPase 1 protein recognize and binds to the nucleocapsids of invading viruses and prevents the intracellular transport of the nucleocapsids into the cell nucleus, which ultimately leads to an early block of the viral transcription and replication (Patzina, Haller & Kochs, 2014). In our analyses, there was an increase in *IFIH1*, *SAMHD1*, *MX1*during viral infection, which might be due to the early stage of infection.

E3 ubiquitin ligase deltex 3L is another protein induced by interferon and it forms complex with poly (ADP-ribose) polymerase PARP9 and targets both host histone H2BJ to promote ISG expression and also Viral 3C protease to disrupt viral assembly in both nucleus and cytoplasm (Zhang et al., 2015). Phospholipid scramblase (PLSCR1)—a multiply palmitoylated, and lipid-raft-associated endofacial plasma membrane protein are induced by INF. Upon induction, newly synthesized PLSCR1 is not palmitoylated, and so it easily enters into nuclei via importin α/β nucleopore transport where it binds to DNA and induces the expression of certain critical antiviral genes, including ISG15, ISG54, p56, and guanylate binding proteins (Wiedmer et al., 2003; Dong et al., 2004). Serine protease inhibitors (serpins) being elements of the innate immune system and belong to the largest and most diverse family of protease inhibitors. Serpins reportedly interfere with viral replication at both the entry and the reverse transcription stages (Asmal et al., 2012). The gene SERPINE encodes plasminogen activator inhibitor (PAI) and has been reported that PAI-2 expression significantly reduced the surface expression of the virus receptor molecules DAF, CAR, and ICAM-1 and thereby inhibits the binding of Virus and exhibits antiviral effect (Congote, 2006). In our study, we observed that there was an increase in the level of serpine in virus-infected cells. Argininosuccinate synthase (ASS) catalyzes the reversible ATP-dependent ligation of citrulline and aspartate to generate argininosuccinate. Importantly, ASS physically interacts with bacterial lipopolysaccharides and lipid A and inactivates their biological activities (Satoh et al., 2006). However, much detailed mechanism was not investigated and in our study, along with ASS1, there was an increased in E3 ubiquitin ligase deltex 3L, PLSCR1 and serpin were increased, which might be one of the antiviral effects induced by the host cell during the early stage of viral infection.

Besides antiviral proteins in the host system, a few pro-viral proteins were consistently increased at the mRNA level involved in virus-mediated infection, and they include *TP*, *Cox-2*, complement 1s and factor B, *IFI44*, *XAF4*, *CXCL3*. Thymidine phosphorylase (TP) is a potent angiogenic factor and a putative marker of cellular oxidative stress and is upregulated in patients of HBV and HCV infected liver tissue with an early event, and it becomes more prominent as the disease progresses to cirrhosis. However, further research has not been carried out on TP during viral infection (Mimidis et al., 2005). Endothelin-1 (ET-1) has been shown to exhibit several physiologic functions, including salt and water homeostasis, vascular tone, and inflammation. Increased cytokines production during viral infection would increase the levels of endothelin-1 in the cells (Bouallegue, BouDaou & Srivastava, 2007). Upon stimulation, ET1 is reported to increase the production IL-6, TNF-alpha, ICAM, VCAM, and e-selectin, which would potentiate inflammation (Christ-Crain, Schuetz & Müller, 2008). Thus, the use of endothelin blockers might effectively reduce the virus-induced lung inflammation.

Prostaglandin-endoperoxide synthase 2 (*PTGS2*) or Cyclo-oxygenase2 (Cox-2) is an inducible pro-inflammatory enzyme. The structural proteins from the SARS-CoV reported to induce the expression of COX-2 in vitro (Liu et al., 2007), and there by increased expression of PGs in the blood of SARS-CoV-infected individuals (Lee et al., 2004). Additionally, the activity of COX might be required for efficient entry and also for an initial step in RNA replication and they suggested that this could be targeted for anti-CoV therapy (Raaben et al., 2007). COX-2 also reported to play a crucial role in limiting the anti-viral cytokine/interferon response to viral infection and thereby thus use of effective COX-2 selective inhibitor during early viral infection, may enhance and/or prolong endogenous interferon responses, and thereby might increase anti-viral immunity (Kirkby et al., 2013). In our analyses, we observed that COX-2 expression was increased in virus-infected cells.

The complement is an important element of innate immunity that functions to recognize and eliminate invading microbes. In our current study, we observed that increased expression of classical pathway protein C1s and alternative pathway protein Factor B during viral infection. In a SARS-CoV-infected mice study, it was reported that complement activation results in immune-mediated damage in the lung of C3 deficient mice, which suggested that inhibition of the complement pathway might be an effective therapeutic strategy to inhibit coronavirus-mediated respiratory diseases (Gralinski et al., 2018). Chemokines being a family of small, secretory proteins are expressed in constitutively or in an inducible manner. The important role of chemokines is to attract leukocytes to sites of infection/inflammation and has been reported that chemokines CXCL2 and CXCL3 are increased in the herpes virus, arenavirus, and rhabdovirus (Melchjorsen, Sørensen & Paludan, 2003) and thus targeting CXCL2/3 could be an effective therapeutic target during viral infection.

IFI44 is a cytoplasmic protein and a type I IFN-induced protein and is upregulated upon a variety of virus infections. Generally, ISGs display antiviral functions; however, IFI44 has been reported to negatively modulate antiviral responses induced by multiple viral systems. IFI44, by inhibiting activates NF-κB which migrates to the nucleus and activates pro-inflammatory cytokines (DeDiego et al., 2014). IFI44 is responsible for the pathogenesis of some viruses, including coronaviruses (DeDiego et al., 2019). XAF1 is an apoptosis-promoting factor by binding to XIAP, which involves in caspase suppression and inhibits cell death. It has been reported that XAF1 promotes apoptosis in DENV2-infected HUVECs by binding to XIAP and also collaborates with TNF-related apoptosis-inducing ligand (TRAIL)-induced cell death (Leaman et al., 2002). In our analysis, we have observed that XAF1 and IFI44 protein in virus-infected cells, which could be targeted against viral infection.

At present, the drug-repurposing approaches on SARS-CoV-2 based on different host factor is being carried out (Zhou et al., 2020; Ge et al., 2020). We have used host transcriptome based pro-viral factors for the drug repurposing against SARS-CoV-2 early infection conditions. Among the 31 up-regulated genes, the pro-viral host factors in SARS-CoV-2 infection were identified by literature-survey, which includes, *TYMP*, *PTGS2*, *C1S*, *CFB*, *IFI44*, *XAF1*, *CXCL2* and *CXCL3*. The genes, *TYMP*, *C1S* and *CFB*, were found to be unique in SARS-CoV-2 infection. The other genes, *PTGS2*, *IFI44*, *XAF1*, *CXCL2* and *CXCL3*, reports the up-regulation tendency in the other respiratory virus infections as well. When considering the pro-viral factors identified from the study for the therapeutic strategies against SARS-CoV-2, drugs targeting PTGS2 are found to be of potential use and are obtained from the DrugBank search. We could notice that six anti-inflammatory drugs targeting PTGS2, lenalidomide, celecoxib, tenoxicam, meclofenamic acid, sulfasalazine, and loxoprofen can induce anti-correlated expression signature with the pro-viral factors and can be considered for the therapeutic purpose of SARS-CoV-2 infection. Importantly, the PTGS2 inhibiting drugs celecoxib and loxoprofen already reported to be useful for the treatment of viral infection. The drug, celecoxib, was shown to decrease inflammatory gene expression in the context of TC-83 virus infection (Risner et al., 2019). The drug, loxoprofen found to be useful for patients with acute upper respiratory tract infection, including those with influenza infection (Azuma et al., 2011). Importantly, the PTGS2 inhibitors reported in the study, indomethacin, naproxen, ibuprofen, and thalidomide are currently under the clinical trials for treating COVID-19.

The approved drug molecules obtained from CMap analysis are from different therapy area including the menopausal hormone therapy (estrone), antiseptic (hexylresorcinol), anti-anxiety agent (pentobarbital), antihypertensive agent (nitrendipine), analgesic (phenazopyridine), neurological (heraclenol), anxiety disorder (alprazolam), dopaminergic activity (bromocriptine) and anticoagulant activity (WT-171 (Vorinostat)). However, anti-anxiety agent, pentobarbital is reported to have various adverse effects hepatotoxicity, laryngospasam, anemia, bradycardia and respiratory depression (AbouKhaled & Hirsch, 2008). Anti-viral activity has been reported for many of the experimental or investigational compounds identified from our study. The study by Nukuzuma et al. (2009) reports that the 5HT(2A)R antagonist, ritanserin have an inhibitory effect on human polyomavirus, JCV infection and reproduction. JAK3 inhibition is another anti-viral strategy that is found to block multiple cytokines and protect against a super inflammatory response (Xu et al., 2012). Tipifarnib may inhibit the prenylation step of the Hepatitis Delta Virus (HDV) replication (Lempp & Urban, 2017). Anti-viral effect against species associated with upper respiratory tract infection (URTIs) or known to cause acute sore throat observed with the drug hexylresorcinol (Shephard & Zybeshari, 2015). Alprazolam and dilazep found to be effective in Influenza Virus Infection and HIV, respectively, and danaprevir is a hepatitis C virus protease inhibitor (Freire-Garabal et al., 1993; Zeng et al., 2014; Gane et al., 2014). Apart from that, the calmodulin antagonist and topoisomerase I inhibitor have reported antiviral activity against the Dengue virus and HIV infection (Bautista-Carbajal, 2017; Showalter & Blair, 2016). Therapeutically targeting the kinases such as PI3K and P38 kinase found to be useful in viral infection (Wu et al., 2015, Griego et al., 2000). The proposed compounds from the study can be considered for the validation experiments.

## Conclusions

In summary, we have used publicly available human host gene expression profiles in early infection conditions of SARS-CoV-2 and other respiratory infection viruses and identified the important host factors in SARS-CoV-2 infection. Considering the consistent expression signature and literature evidence, we could identify 31 upregulated host factors, and out of them, eight are pro-viral factors in SARS-CoV-2 infection. The study is an effort to identify repurposed drugs for the treatment of SARS-CoV-2 infection, considering the pro-viral host factors. The connectivity map based repurposing proposed twelve compounds with evident antiviral activity from the literature survey. Apart from that, we could propose that inhibition of PTGS2 can be a potential therapeutic strategy for viral infection, and the proposed six approved PTGS2 inhibitor drugs can be repurposed for the treatment of SARS-CoV-2 infection. The study is based on the presently available dataset in public domain databases and may fluctuate based on the sample selection in the analysis. Therefore, we have considered strict criteria of consistency in expression patterns and evidence from the literature in the selection of host factors for repurposing studies. The rapid therapeutic recommendations from this computational approach can be repeated as more RNA-Seq datasets become available and could be done on protein-level and need to be validated by wet-lab experiments and clinical trials.

## Supplemental Information

10.7717/peerj.9357/supp-1 Supplemental Information 1 Tables S1-S4. Table S1: List of GEO datasets on host transcriptome profiles of different respiratory infection viruses obtained for the study
Table S2: List of significantly differentially expressed genes from the meta-analysis of 24 hr infection of different respiratory infection virus compared with mock control infection
Table S3: Significantly DEG in SARS-Cov2 vs Mock in A549 and NHBE cells
Table S4: Functional Enrichment of the 28 differentially expressed genes

10.7717/peerj.9357/supp-2 Supplemental Information 2 The effect of batch-effect adjustments using the Network Analyst tool. (A) PCA-3D plots (B) Density plots

10.7717/peerj.9357/supp-3 Supplemental Information 3 PRISMA checklist.

## Additional Information and Declarations

### Data Availability

The following information was supplied regarding data availability: All the data came from public domain databases. Details of raw data and the analysis results are available in the Supplemental Files.
