# Supplementary figures and images for: Host transcriptome-guided drug repurposing for COVID-19 treatment: a meta-analysis based approach
Source: PeerJ. 2020 Jun 10;8:e9357. doi: 10.7717/peerj.9357 (PMC7293190; doi:10.7717/peerj.9357)

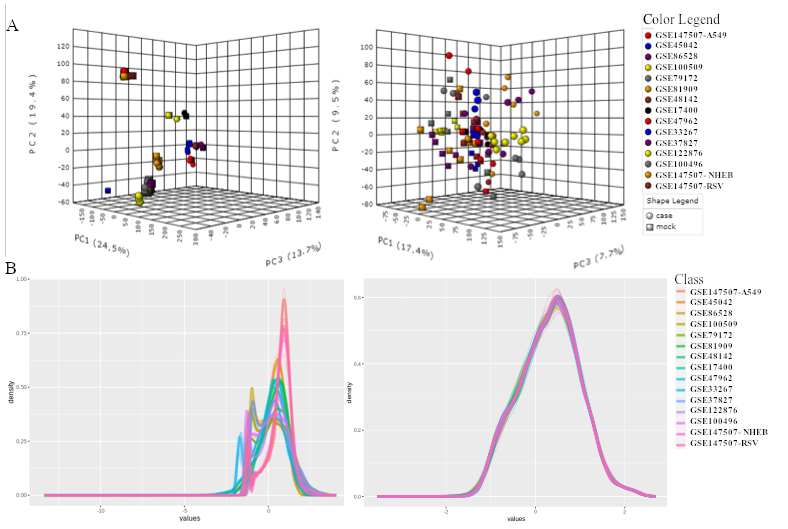

Supplement: Supplemental Information 2 — (A) PCA-3D plots (B) Density plots [file peerj-08-9357-s002.png]
